# Advances in the Management of Early-Stage Triple-Negative Breast Cancer

**DOI:** 10.3390/ijms241512478

**Published:** 2023-08-05

**Authors:** Prarthna V. Bhardwaj, Yue Wang, Elizabeth Brunk, Philip M. Spanheimer, Yara G. Abdou

**Affiliations:** 1 Division of Hematology-Oncology, University of Massachusetts Chan Medical School—Baystate, Springfield, MA 01199, USA; 2 Department of Pharmacology, University of North Carolina at Chapel Hill, Chapel Hill, NC 27599, USA; 3 Curriculum in Pharmacology, University of North Carolina at Chapel Hill, Chapel Hill, NC 27599, USA; 4 Department of Chemistry, University of North Carolina at Chapel Hill, Chapel Hill, NC 27599, USA; 5 Integrative Program for Biological and Genomic Sciences, University of North Carolina at Chapel Hill, Chapel Hill, NC 27599, USA; 6 Lineberger Comprehensive Cancer Center, UNC Chapel Hill, NC 27599, USA; 7 Computational Medicine Program, UNC Chapel Hill, NC 27599, USA; 8 Department of Surgery, University of North Carolina at Chapel Hill, Chapel Hill, NC 27599, USA; 9 Division of Oncology, University of North Carolina at Chapel Hill, Chapel Hill, NC 27599, USA

**Keywords:** breast cancer, breast surgery, early-stage TNBC, immunotherapy, neoadjuvant chemotherapy, PARP inhibitors, triple-negative breast cancer, targeted therapy

## Abstract

Triple-negative breast cancer (TNBC) is a subtype of breast cancer with both inter- and intratumor heterogeneity, thought to result in a more aggressive course and worse outcomes. Neoadjuvant therapy (NAT) has become the preferred treatment modality of early-stage TNBC as it allows for the downstaging of tumors in the breast and axilla, monitoring early treatment response, and most importantly, provides important prognostic information that is essential to determining post-surgical therapies to improve outcomes. It focuses on combinations of systemic drugs to optimize pathologic complete response (pCR). Excellent response to NAT has allowed surgical de-escalation in ideal candidates. Further, treatment algorithms guide the systemic management of patients based on their pCR status following surgery. The expanding knowledge of molecular pathways, genomic sequencing, and the immunological profile of TNBC has led to the use of immune checkpoint inhibitors and targeted agents, including PARP inhibitors, further revolutionizing the therapeutic landscape of this clinical entity. However, subgroups most likely to benefit from these novel approaches in TNBC remain elusive and are being extensively studied. In this review, we describe current practices and promising therapeutic options on the horizon for TNBC, surgical advances, and future trends in molecular determinants of response to therapy in early-stage TNBC.

## 1. Introduction

Triple-negative breast cancers (TNBC) are a diverse subgroup of breast cancer defined by the absence of estrogen receptor (ER), progesterone receptor (PR), and human epidermal growth factor receptor 2 (HER2) expression. The dynamic tumor microenvironment in TNBC is associated with resistance to chemotherapy, aggressive course, frequent recurrence, and worse prognosis [1]. Nearly 20% of patients with TNBC harbor a breast cancer susceptibility gene (BRCA) mutation, particularly BRCA1, compared to 6% of all breast cancers associated with a BRCA mutation [2]. Traditionally, chemotherapy has been the only systemic treatment option in early-stage TNBC; however, the more recent introduction of novel agents, including immunotherapy and PARP inhibitors, is altering the treatment paradigm for this disease. Given significant inter- and intratumor heterogeneity in TNBC [3,4,5], studies are being pursued to understand and address this biological complexity to tailor therapeutic strategies appropriately.

In this review, we will describe current practices in the medical and surgical management of early-stage TNBC. We will also focus on current treatments in the pipeline that are predicted to contribute to upcoming progress in the field by integrating clinical and molecular characteristics of TNBC.

Molecular Subtypes of TNBC

All breast cancers are characterized by intrinsic molecular subtypes when gene expression profiling is undertaken, including Luminal A, Luminal B, HER2-enriched, basal-like, normal-like, and Claudin low subtypes [6]. Intrinsic subtypes help provide predictive information on response to neoadjuvant therapy (NAT) in breast cancers but are usually inconsistent in the triple-negative subtype [7]. Disagreements between classifications and a high proportion of specimens that cannot be classified are some reasons why intrinsic subtypes are deemed to be less reliable biomarkers of response [8]. Several molecular classifications of TNBCs have been proposed and identified, but the Lehmann/Pietenpol subtypes are most frequently utilized, as described in Table 1. Lehmann et al. identified six TNBC subtypes through gene expression analyses and identified the unique signaling pathways that each subtype may be enriched in. This resulted in cell line models that have facilitated preclinical experiments to define responses to selected targeted therapies in vivo [9].

Other less commonly used classifications include Burstein subtypes, FUSCC classification, Integrative Clusters, and Prado-Vazquez classification [11,12,13,14,15].

Combining molecular knowledge with patient management is the core principle of precision medicine. Still, the lack of clinically actionable biomarkers of response within TNBC has led to the use of a “one-size fits all” strategy that has not necessarily been successful.

## 2. Systemic Therapy—New Insights

Neoadjuvant systemic therapy (NAT) is now the standard of care for most early-stage TNBC. Advantages to this approach include downstaging of tumors resulting in improved rates of breast-conserving surgery, downstaging of axilla resulting in reduced axillary dissection, and tailoring of adjuvant treatment based on treatment response [16]. NAT also provides a window of opportunity between diagnosis and surgical resection for translational research and assessment of biomarkers [17]. Furthermore, this approach can predict survival based on the status of pathological complete response (pCR), defined as the absence of residual invasive disease in the breast or lymph nodes at the time of surgery, allowing for tailoring of subsequent therapies. Achieving pCR is an important goal in TNBC as it is associated with improvement in long-term outcomes, including event-free survival (EFS) and overall survival (OS) [18,19,20,21].

### 2.1. Advances in Chemotherapy

Cytotoxic chemotherapy is currently considered the main systemic treatment for early-stage TNBC, yet the ideal treatment regimen remains unclear. Although the efficacy of a taxane-anthracycline-based regimen has been established in this disease [22], the role of adding platinum remains controversial. Several trials looked at the addition of carboplatin in the neoadjuvant setting (Table 2) [23,24,25]. Although the CALGB 40,603 trial did not reveal any survival benefit with the addition of carboplatin [8], an improved disease-free survival (DFS) and EFS was noted in the GeparSixto (HR 0.56; 95% CI 0.34 to 0.93; *p* = 0.022) and the BrighTNess (HR 0.57, 95% CI 0.36 to 0.91; *p* = 0.018) trials, respectively, for patients treated with platinum [25,26]. However, none of these studies demonstrated an OS benefit with this approach. Furthermore, a meta-analysis, including 2109 patients from nine trials who had received NAT with platinum-based versus platinum-free chemotherapy, showed that platinum-based therapy increased pCR rates by approximately 10–15%. However, this improvement in pCR did not translate into a survival benefit [27]. Therefore, the benefit of platinum-based therapies remains unclear and comes at the cost of increased hematologic toxicities and early treatment discontinuations in early-stage TNBC.

Another important consideration for chemotherapy administration is the benefit of dose-dense scheduling. This was noted in the AGO Phase III Study, where improved ten-year OS was noted with dose-dense administration (69% vs. 59%; HR 0.72; 95% CI 0.60 to 0.87; *p* = 0.0007) [31]. Correspondingly, a meta-analysis of 26 randomized clinical trials by the Early Breast Cancer Trialists’ Collaborative Group (EBCTCG) revealed a moderate reduction in 10-year risk of recurrence (31.4% vs. 28.0%; RR 0.86, 95% CI 0.83 to 0.92) and death from breast cancer (18.9% vs. 21.3%; RR 0.87, 95% CI 0.83 to 0.92) without increasing mortality from other causes with a dose-dense approach to NAT [32]. Although the benefit of dose-dense anthracyclines appears to be clear, more studies are needed to establish the additional benefit seen with dose-dense versus weekly paclitaxel.

Other endeavors to further tailor therapy for early-stage TNBC have involved the addition of sequential capecitabine in the adjuvant setting following NAT and surgery in patients with residual disease. In the Create-X trial, patients who did not achieve pCR were randomized to adjuvant capecitabine for 6–8 cycles or a control group with no further therapy. This strategy resulted in prolonged DFS as well as OS (78.8% vs. 70.3%, HR 0.52; 95% CI 0.30 to 0.90]) [33]. Similarly, the SYSUCC-001 trial demonstrated an improvement in the 5-year DFS with the use of one year of adjuvant capecitabine (82.8% vs. 73.0%, HR 0.64; 95% CI 0.42 to 0.95; *p* = 0.03) although there was no improvement in OS noted in this study [34].

On the contrary, the GEICAM/2003-11 trial did not show a statistically significant increase in DFS with adjuvant capecitabine [35]. This difference in results raises considerations regarding the influence of ethnic differences on the biology of TNBC as Create-X and SYSUCC-001 enrolled a predominantly Asian population known to metabolize fluoropyrimidines efficiently, whereas GEICAM accrued patients from Europe and South America. A pre-planned analysis of the GIECAM study demonstrated that the non-basal TNBC cohort derived the most benefit from receiving capecitabine, thus indicating the need for detailed investigation into the intrinsic subtypes of TNBC who would most benefit from capecitabine [35].

### 2.2. Immunotherapy

Despite lacking canonical targets for biologic treatment, TNBC may demonstrate a higher tumor mutational burden (TMB), higher PD-L1 expression, and more tumor-infiltrating lymphocytes compared to other subtypes [36,37,38,39], which are associated with an increased response to immunotherapy [40]. The landscape of early-stage TNBC has changed with the recent approval of immune checkpoint inhibitors (ICIs) combined with chemotherapy. ICIs were initially approved for metastatic, PD-L1-positive TNBC based on improvements in outcomes [41,42]. Evidence suggested a superior efficacy of ICIs when administered early in TNBC due to the progression of immune escape mechanisms during the advancement of disease [43,44]. This idea formed the rationale for clinical trials in early-stage breast cancer with the aim of providing ICIs earlier in the disease course prior to surgery.

The KEYNOTE-522 (KN522) was pivotal in bringing ICI to the frontline treatment of early-stage TNBC. In this study, patients were randomized to receive neoadjuvant therapy with four cycles of pembrolizumab or placebo plus paclitaxel and carboplatin followed by an additional four cycles of pembrolizumab or placebo followed by anthracycline-cyclophosphamide [45]. Patients then went on to receive adjuvant pembrolizumab or placebo. There was an improvement in pCR (64.8% vs. 51.2%) and EFS (84.5% vs. 76.8%, HR 0.63; 95% CI 0.48 to 0.82, *p* < 0.001) in the chemo-immunotherapy arm [46]. Across all treatment phases, the incidence of grade 3 or higher treatment-related adverse events was similar (78% vs. 73%) [45,46]. Based on these results, the Food and Drug Administration (FDA) approved the use of pembrolizumab in combination with chemotherapy for high-risk early-stage TNBC as neoadjuvant treatment as well as monotherapy in the adjuvant setting.

#### 2.2.1. Other Checkpoint Inhibitors in Breast Cancer

Several other randomized trials have investigated the addition of ICIs to neoadjuvant chemotherapy in early-stage TNBC (Table 3). Impassion031 also showed improved pCR when atezolizumab was added to anthracycline-based chemotherapy (58% vs. 41%, 95% CI 6 to 27; *p* = 0.0044), especially in patients with positive PD-L1 vs. PDL1 negative (69% vs. 49%) [47]. Despite similar results to KN522, atezolizumab for early-stage TNBC was withdrawn in Europe based on the impression that the benefits of atezolizumab did not outweigh the risks in this population based on a primary endpoint of pCR alone. In contrast to KN-522 and IMpassion031, the NeoTRIP failed to demonstrate a difference in pCR with the use of atezolizumab in combination with chemotherapy in the neoadjuvant setting (48.6% vs. 44.4%, OR 1.18; 95% CI 0.74 to 1.89; *p* = 0.48) [48]. This discrepancy in outcomes between NeoTRIP and prior studies is not clear. One possible explanation is that there were fewer patients with locally advanced or stage III TNBC in IMpassion031 and KN522 compared to the NeoTRIP study (25%, 25%, and 49%, respectively). Another possible reason is the choice of chemotherapy that included sequential neoadjuvant regimens, including an anthracycline combination in the prior two studies compared to an anthracycline-free neoadjuvant regimen in this study.

Notably, the GeparNuevo study evaluating durvalumab/chemotherapy combination in the neoadjuvant setting showed an improvement in 3-year DFS (85.6% vs. 77.2%, *p* = 0.036) but no statistical differences in pCR rates [49]. However, the multivariable analysis revealed a durvalumab effect independent of pCR effect. A unique aspect of this study was the “window of opportunity” cohort who received two weeks of durvalumab alone before the commencement of chemotherapy. Interestingly, patients in this cohort experienced greater pCR benefits with Durvalumab.

Overall, these data have established the role of neoadjuvant ICIs in higher-risk early-stage TNBC. More studies are vital to risk-stratify these patients to optimize treatment recommendations while minimizing toxicity. Table 3 summarizes major clinical trials with immunotherapy that are currently pending or have resulted in the neoadjuvant and adjuvant setting.

#### 2.2.2. Challenges and Future Directions

The approval of ICIs in early-stage TNBC has raised several questions. Despite the success of pembrolizumab in the KN522 study, no biomarker has predicted the pattern of response in patients with early-stage TNBC including PD-L1 level, unlike the metastatic setting where PD-L1 level was somewhat predictive. A consistent benefit of ICI was noted regardless of tumor size, age, carboplatin schedule, and performance status. Although standard parameters have not so far helped in selecting patients for immunotherapy, novel markers, including circulating tumor DNA (ctDNA), have emerged as a relevant prognostic marker in breast cancer [55,56] and their clinical value is now being investigated as potential predictive biomarkers of ICIs and treatment resistance to maximize the personalized benefit of ICIs.

Furthermore, the combination of pembrolizumab with prior standard therapies remains a challenge. Adjuvant capecitabine in patients with residual disease following NAT [33] and adjuvant olaparib in high-risk HER2-negative patients with BRCA1/2 mutations [57] were not studied in the KN522 trial, where all patients received pembrolizumab alone as adjuvant therapy irrespective of residual disease at surgery. To our knowledge, there are no data on the efficacy and safety of concurrent or sequential capecitabine or olaparib when combined with pembrolizumab in the adjuvant setting for patients with residual disease post-NAT in early-stage TNBC. Although, safety data have been reported for these combinations (capecitabine plus pembrolizumab; olaparib plus pembrolizumab) in the metastatic setting. Based on best judgment and the currently available framework, there is a proposition to use pembrolizumab with capecitabine in patients with residual disease and pembrolizumab with olaparib for high-risk BRCA-mutant patients [58].

Next, it is also important to clarify if pembrolizumab is truly essential and efficacious in the adjuvant setting, as ICIs come with the risk of immune-related adverse effects beyond the toxicities of traditional chemotherapy and increased economic burden due to the high cost of these medications. For instance, the GeparNuevo study only incorporated ICI in the neoadjuvant setting and still demonstrated improved EFS, suggesting that ICIs may not need to be continued in the adjuvant setting [48]. In that regard, OptimICE-pCR is designed to study clinical outcomes, including invasive DFS of adjuvant pembrolizumab, compared to no therapy in early-stage TNBC patients who have received NAT with pembrolizumab and achieved pCR [59,60].

Furthermore, despite adjuvant pembrolizumab in KN522, EFS was only 67.4% in patients with residual disease at surgery, with worse EFS in patients with higher residual cancer burden (26.2% in patients with RCB-3) [61], highlighting the need to find alternative treatment strategies in these patients. OptimICE-RD [NCT05633654] and the SASCIA trial [NCT04595565] are evaluating the addition of sacituzumab govitecan in patients with residual disease [62,63].

Finally, there are several ongoing efforts to evaluate the safety and efficacy of other immunotherapeutic approaches in TNBC, including vaccine therapies, T-cell regulatory immunomodulators, and chimeric antigen receptor-modified T (CAR-T) cell therapy in all stages of TNBC [64]. Although several of these therapies have been studied in phase I and phase II studies in the metastatic setting, they are being incorporated into the treatment of early-stage TNBC as well. Of note, oncolytic virus therapy is a modality of treatment that has shown promising efficacy in early-stage breast cancer. In a phase II clinical trial, patients with early-stage TNBC received intratumoral Talimogene-laherparepvec (T-VEC), an oncolytic virus, alongside neoadjuvant chemotherapy; 45.9% of patients achieved a residual cancer burden index (RCB) of 0 (corresponding to pCR), whereas 65% had RCB-I [65].

### 2.3. Targeted Agents

**Figure 1:**
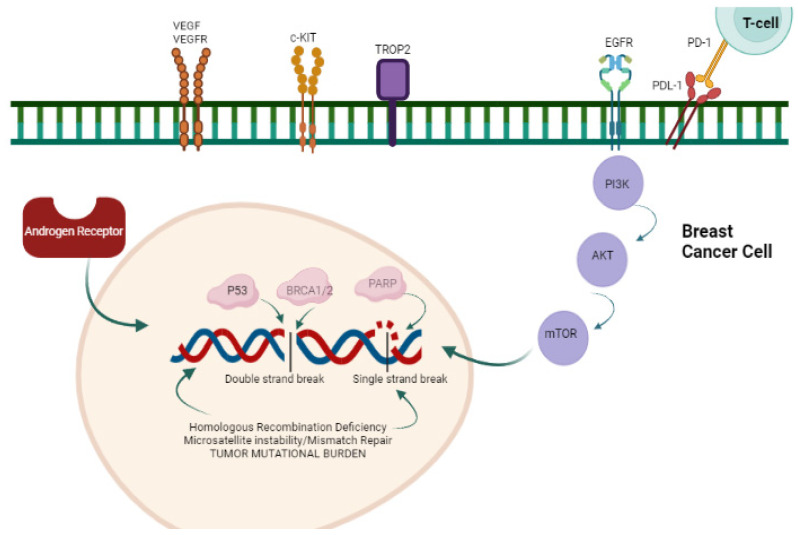
Major abnormal signaling pathways with potential therapeutic targets in TNBC (*created using BioRender.com*).

With the development of next-generation sequencing (NGS), novel targets have been identified for patients with metastatic breast cancer but are still being explored in the early-stage setting. TP53 mutations are the most frequent mutations (60–70%) commonly in basal-like TNBC, followed by PIK3CA (~10%) seen often in LAR TNBC [4]. Other mutations occur at a low (1–5%) to very low (<1%) frequency, some of which can be targetable, like ERBB2 and BRAFV600E, through currently available therapies. Figure 1 depicts major therapeutic targets in TNBC.

#### 2.3.1. PARP Inhibitors (PARPi)

TNBCs are often deficient in DNA Damage Response (DDR) pathways and display high chromosomal instability [4]. Poly ADP-ribose polymerase (PARP) is vital for double-strand break (DSB) repairs through the homologous recombination pathway [66]. In tumors harboring a defect in the homologous recombination pathway, inhibition of PARP enzymes leads to the accumulation of unpaired damages leading to cell cycle arrest and death. Following success in the metastatic setting, PARP inhibitors have now emerged in early-stage disease. Specifically, olaparib was approved by the FDA in March 2022 as adjuvant therapy for high-risk HER2 negative breast cancer with germline BRCA1/2 mutations based on the OlympiA trial, which demonstrated improvement in DFS (85.9% vs. 77.1%, HR 0.68; 95% CI 0.50 to 0.91; *p* = 0.0091) and OS (HR 0.68; 98.5% CI 0.47 to 0.97; *p* = 0.009) [57,67]. Based on this study, expert consensus favors the use of olaparib over capecitabine in germline BRCA carriers with high-risk TNBC and residual disease following NAT, although there is no direct comparison between the two.

In the neoadjuvant setting, addition of veliparib, which has weak PARP-trapping activity, to platinum/paclitaxel showed improved pCR rates in I-SPY2 compared to single-agent paclitaxel (51% vs. 26% in TNBC) whereas pCR rates were comparable in the BrighTNess trial between veliparib/platinum/paclitaxel and platinum/paclitaxel (53% vs. 58%, *p* = 0.36) [23,68]. This suggests that the increase in pCR seen in I-SPY2 could have been related to platinum rather than veliparib and that there may not be a synergistic effect between platinum and PARP inhibitors.

Subsequently, the NEOTALA study tested talazoparib monotherapy preoperatively in BRCA1/2 mutated HER2 negative breast cancers showing pCR rates of 49%, which is numerically comparable to those receiving neoadjuvant chemotherapy [69].

In summary, neoadjuvant treatment with PARP inhibitor in BRCA-mutated TNBC may influence pCR but there is no additional benefit in combining it with a platinum-containing chemotherapy. None of the studies utilizing PARPi (Table 4) have shown compelling evidence to currently use them in the neoadjuvant setting as a standard of care. More research is required in terms of survival benefits, safety, and optimal patient population before this can be accomplished.

#### 2.3.2. PIK3CA/AKT1/PTEN Pathway

PI3K/AKT/mTOR pathway-associated mutations may be seen in TNBC. Although there are no currently approved therapies in early-stage TNBC, this pathway has been explored for potential therapeutic benefit [72]. It is most commonly activated by PIK3CA mutations (9–18%), loss of PTEN (35%) or INPP4B (30%), and amplifications of PIK3CA (43%) [72,73]. PIK3CA mutations are more common in mesenchymal or LAR subtypes [9,73]. Pre-clinical studies have demonstrated the role of PI3K/mTOR inhibitors in producing a cytostatic effect, but, in combination with chemotherapy, resulted in cell death [74]. Results from a phase II trial [NCT04216472] evaluating the combination of alpelisib and nab-paclitaxel in anthracycline refractory TNBC with PIK3CA or PTEN alterations are awaited.

AKT1, AKT2, and AKT3 are closely related proteins that have downstream effects and are potentially targetable. Ipatasertib, an AKT inhibitor, when added to neoadjuvant chemotherapy in the FAIRLANE study, did not significantly increase the pCR rate in patients with early-stage TNBC [75]. However, the antitumor effect of ipatasertib seemed to be more noticeable in patients with PIK3CA/AKT1/PTEN alterations based on unconfirmed clinical responses.

Everolimus, an mTOR inhibitor, has been investigated in combination with cisplatin/paclitaxel [76], and with docetaxel/5-FU/epirubicin/cyclophosphamide [77] in neoadjuvant treatment for TNBC. There were no improvements in response rates in either of the studies.

In summary, the efficacy of drugs against the PI3K/AKT/mTOR pathway in TNBC has not lived up to the potential observed in the pre-clinical setting. This could be justified by the complex nature of the immune microenvironment and confounding molecular alterations or parallel pathway activation resulting in a resistance mechanism that may make accurate estimation of clinical benefit challenging. Secondly, the best actionable target within this pathway may differ based on mechanisms of pathway activation and escape feedback.

#### 2.3.3. Androgen Receptor (AR) Pathway

AR expression is found in approximately 10–35% of TNBC [78], especially in the LAR subtype. Although enzalutamide monotherapy [79], enzalutamide with PIK3CA inhibitor [80], abiraterone, and bicalutamide [81,82] have shown modest results in advanced TNBC, there are no conclusive data in the early-stage setting. A phase II trial evaluating enzalutamide in combination with paclitaxel in the neoadjuvant setting in AR-positive TNBC is currently underway [NCT02689427].

#### 2.3.4. Receptor Tyrosine Kinase Family (HER2, VEGF)

*HER2:* A small subset of TNBC patients harbor somatic ERBB2 mutations [3]. In an exploratory analysis of a cohort of the I-SPY2 trial treated with neratinib in TNBC, increased EGFR Y1173 (*p* = 0.005) and HER2 Y1248 (*p* = 0.019) phosphorylation were a predictor of pCR [83]. Additionally, neratinib in the neoadjuvant setting demonstrated pCR of 37.5% which increased to 62.5% in patients displaying phosphorylation of HER2 or EGFR [84].

There is increasing evidence that close to 35% of TNBCs may be reclassified as HER2-low [85], which has expanded therapeutic options in this subset. The encouraging results in HER2 low-expressing breast cancer in the metastatic setting observed in the DESTINY-Breast 04 study with the antibody–drug conjugate, trastuzumab deruxtecan, will likely pave the way for the use of these agents in the early-stage setting [86].

*VEGF*: Several studies have assessed the utility of bevacizumab, a monoclonal antibody targeting vascular endothelial growth factor A (VEGF-A) like the ARTemis and GeparQuinto; they demonstrated improved pCR rates with the addition of bevacizumab to chemotherapy especially in TNBC patients in the neoadjuvant setting [25,87]. However, this did not translate into an OS benefit in either of the studies [88]. Similarly, CALGB 40,603 showed improved pCR in the breast with the addition of bevacizumab but not in the axilla and had no impact on OS [8]. In the adjuvant setting, adding bevacizumab to anthracycline or taxane-based chemotherapy did not show a difference in invasive DFS or OS per the BEATRICE trial [89]. Hence, VEGF inhibitors are not currently considered standard-of-care therapies.

Several other targets, including EGFR, FGFR, TROP-2, JAK/STAT3, and CDK4/6 pathways are being studied in early-stage TNBC. Of particular importance is sacituzumab govitecan, an antibody–drug conjugate that has demonstrated success in the metastatic setting and is now being extensively studied in the early-stage setting. Table 5 summarizes some ongoing clinical trials studying targeted agents in the management of early-stage TNBC.

Finally, several other novel molecular biomarkers are being identified in TNBC whose therapeutic benefits are yet to be explored fully including Axl [90], Wnt pathway [91], and paraoxanase-2 [92].

## 3. Advances in Surgery

Surgical management of breast tumors has undergone significant advances since the initial description by Halsted in 1898. A better understanding of disease biology and advances in systemic and radiotherapy have allowed for the de-escalation of surgery without compromising oncologic outcomes. Although no surgical techniques are specific to TNBC, advances in chemotherapy and immunotherapy have led to changes in timing and more importantly, the extent of surgery. Therefore, advances in surgery for TNBC are in principle safe de-escalation and omission of surgical procedures.

Over the past few decades, NAT regimens have been increasingly adopted for patients with TNBC. As discussed previously, the use of NAT provides prognostic information from tumor response assessed on surgical pathology, which is used to stratify patients for additional adjuvant therapy. Additionally, NAT is well established to increase patient eligibility for breast conservation and more recently is being used to downstage the axilla to avoid axillary lymph node dissections (ALND); these effects are most pronounced for TNBC compared to other subtypes [93,94]. ALND is associated with significant risks of neuropathy, lymphedema, and arm dysfunction [95,96]. Despite early concerns about the potential to miss residual nodal disease after NAT, several groups have demonstrated the ability to identify residual nodal disease with acceptable false negative rates in patients with clinically node-positive disease who are treated with NAT. The ACOSOG Z1071 trial showed that in clinically node-positive patients treated with NAT, the false negative rate of outback sentinel lymph node biopsy (SLNB) was under 10% with dual tracer and three or more recovered nodes [97], which was similar to the SENTINA trial which showed a reduction in false negative rate with increased nodal recovery [98]. The MD Anderson group adopted an approach of retrieving the previously biopsied clipped node, which demonstrated improved performance over the sentinel node alone [99]. This has been independently validated in another study [100]. Although performance metrics with the retrieval of the clipped node are improved over SLNB alone, oncologic safety of axillary staging using SLNB alone has been shown. In a prospective observational study of patients with clinically node-positive breast cancer treated with NAT where ALND was not completed if sentinel nodes were negative and SLNB was performed with dual tracer and a minimum of three nodes recovered, the nodal recurrence rate was under 1% at a median follow up of 40 months [101].

The accuracy of axillary staging after NAT and the potential to avoid ALND in patients with good response to systemic therapy has led to significant interest in optimizing systemic regimens to maximize nodal clearance rates. In the BrighTNess trial, patients were treated with doxorubicin/cyclophosphamide followed by taxol and randomized to the addition of carboplatin, demonstrating increased nodal clearance with the addition of carboplatin [26]. Quickly following BrighTNess, KN522 showed that the addition of pembrolizumab further increased the rate of nodal disease clearance [45]. As rates of nodal clearance increase and thereby increase the prevalence of true negatives, the potential burden of false negatives is depleted; however, establishing a clinically actionable threshold whereby accurate post-chemotherapy axillary staging is no longer necessary, will prove to be challenging.

With improved responses, two important horizons remain for de-escalation of surgery in patients following neoadjuvant chemotherapy. First, as trials demonstrate that microscopic node-positive disease is not a driver of recurrence or survival [95,102], the benefit of routine axillary lymph node dissection for patients with residual node-positive disease after neoadjuvant chemotherapy is being questioned. Concerns remain that this represents a population of patients enriched for chemotherapy-resistant disease who are at increased risk for regional failure if surgical clearance of regional lymph nodes is omitted. Supporting the safety of this approach, regional nodal irradiation is recommended for all patients with known nodal disease prior to neoadjuvant. The Alliance 11,202 trial [NCT01872975] is ongoing to address the benefit of routine ALND in patients with residual node-positive disease after NAT vs. nodal irradiation alone [103]. Second, and perhaps representing the pinnacle of systemic therapy for solid organ tumors, is the question of whether all patients with TNBC treated with NAT need surgery at all. The pCR rate was 65% in KN522, and these high rates of complete response have led investigators to question whether resection is needed for patients with evidence of response. Kuerer et al. reported the omission of surgery in patients with TNBC and HER2-positive breast cancer who had exceptional responses to NAT, measured radiographically and with post-NAT percutaneous biopsies [104]. They found that in 21 patients with TNBC where surgery was omitted, there were no recurrences at a median follow-up of 26 months. Several hurdles remain for the implementation of this strategy, including radiographic follow-up and the significant burden of post-NAT percutaneous biopsies to determine response. Therefore, the success of strategies omitting surgery will rely heavily on predicting which patients are most likely to have complete responses. As improved regimens demonstrate higher pCR rates, the omission of surgery will become increasingly more feasible. This also represents an important area of future investigation, as more precise regimens could help increase the number of patients who can avoid surgery altogether.

**Figure 2:**
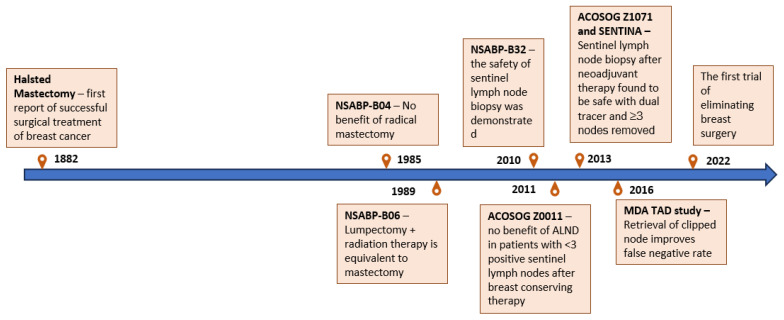
Pivotal advances in the surgical management of breast cancer.

Figure 2 demonstrates the pivotal advances in surgical management of breast cancer.

## 4. Tumor Heterogeneity and the Future of Precision Medicine

Personalized treatment options for TNBC patients are limited by the lack of targeted, patient-specific therapies available in the clinic [105]. A grand challenge in finding these personalized treatments is understanding the extent to which tumor heterogeneity impacts treatment response. Tumor heterogeneity is a “black box” term used in cancer research to indicate how variable a patient’s tumor is in the context of spatial (regional) and molecular variability [106]. The fact that we lack quantitative and systematic methods to explain this variability in concrete terms is a major obstacle impacting treatment outcomes. Distinct subpopulations can be inherently resistant to treatment or primed for developing adaptive resistance [106]. Ultimately, the more heterogeneous a tumor, the higher the likelihood of resistance and poorer prognosis. Each level of heterogeneity contributes significantly to our understanding of how human tumors respond to therapy, but without methods to integrate these levels appropriately and effectively, we are limited in the practical use of this knowledge.

Incorporating tumor heterogeneity into the vision of precision medicine means establishing clinical care that: (1) incorporates knowledge related to intertumoral heterogeneity; (2) assesses how likely a patient will respond to a treatment, given the genetic and molecular profile of their tumor; (3) determines how effective a treatment will be (e.g., how likely the tumor will develop resistance) based on intratumoral heterogeneity of their tumor biopsy. Having an individualized roadmap for each patient that considers comprehensive molecular profiling will enable matching patients to precision clinical trials and, ultimately, a clinician’s ability to match tumors to therapeutics. Such molecular biomarkers of response will enrich responding populations, reduce toxicity, and identify patients needing improved strategies.

Biomarker discovery has benefited from the incredible advances in the fields of genomics and computer science over the last decade. DNA sequencing, transcriptomics, and proteomics datasets are now available for over 11,000 tumors in the Cancer Genome Atlas, of which 1084 are breast cancer tumors and 171 are TNBC subtypes. In addition to tumor data, nine types of multi-omics data have been collected on over 2000 cancer cell lines [107,108], which includes DNA sequencing, functional genomics profiles (transcriptomics, epigenomics, proteomics, metabolomics, etc.), and perturbation screens (drug treatment and CRISPR-mediated knockdown). Of the 2000 cell lines, 31 are TNBC cell lines. Novel single-cell sequencing datasets are also becoming available, both in breast cancer cell lines [109,110,111] and tumors [112]. Where bulk sequencing offers an average picture of cellular activities, single-cell sequencing provides a comprehensive view at single-cell resolution, thereby directly probing intratumoral heterogeneity. Recently, a study has been published demonstrating that both bulk RNA-seq and ssRNA-seq can be simultaneously collected on 26 breast cancer primary tumors [112]. These are exciting advances because RNA when analyzed without other molecular profiling data, can be used for clinically useful predictors of recurrence and response to therapy in breast cancer [113,114,115].

Although this is a significant step in the right direction, there remains a significant opportunity for more global big data analyses that can increase precision in prognostication and prediction of response to therapy, especially in TNBC breast cancer. One of the grand challenges of the field of genomics and systems biology is to expand multi-omic datasets collected for primary breast cancer tumors to include additional data types, such as single-cell RNA sequencing and single-cell ATAC-seq [116]. The technical capacity to perform multiple bulk and single-cell genomics assays on individual pre-treatment breast cancer samples could transform our ability to determine heterogeneity in molecular features that drive therapeutic response and individualize treatment regimens. To accomplish this, enough tissue must be extracted from a biopsy to ensure that high enough quality data can be generated. But what is enough tissue? And what data types will be most informative? These questions demand further testing and will require changes in the way patient data are collected and the way clinical trial experiments are designed.

Incorporating multi-omic approaches to guide diagnosis, treatment, and clinical trial design is the future of precision medicine. It is expected to improve the prediction of response in patients with breast cancer and especially TNBC, due to the ability to tease out intratumoral heterogeneity. Studies incorporating these datasets have the potential to elucidate mechanisms of response and resistance, which can be used to select patients for treatment strategies and uncover more effective treatment strategies for non-responders.

## 5. Conclusions

In conclusion, TNBC remains a heterogenous disease; tremendous progress has been made especially pertaining to early-stage TNBC management. One of the most promising modalities has been the use of immune checkpoint inhibitors; however, questions remain regarding the ideal patients suitable for therapy, optimal chemotherapy partners, the role of postoperative systemic therapy, and biomarkers that predict response early in the treatment course.

Similarly, advances in tumor characterization have allowed for several promising targeted agents, including antibody–drug conjugates on the horizon. However, the optimal way of integrating these agents for treatment combinations is challenging. The TNBC treatment landscape remains an evolving area that represents the crucial relationship between laboratory and clinical research.

## Figures and Tables

**Table 1:** Molecular subtypes adopted from Pinilla et al. [10].

Lehmann et al. [9] Classification *n* = 2347	
Type	Main Molecular Characteristics
Basal-like 1 (BL1)	Enriched in: Cell cycle and proliferation genes (AURKA, AURKB, CENPA, BUB1, TTK, CCNA2, PRC1, MYC, NRAS, PLK1, BIRC5) DNA damage response genes (CHEK1, FANCA, FANCG, RAD54BP, RAD51, NBN, EXO1, MSH2, MCM10, RAD21, MDC1) High Ki-67 mRNA expression
Basal-like 2 (BL2)	Enriched in: Growth factor signaling genes (EGF, NGF, MET, Wnt/β-catenin, IGF1R pathways) Growth factor receptor genes (EGFR, MET, EPHA2) Myoepithelial markers (TP63 and MME or CD10)
Immunomodulatory (IM)	Enriched for gene ontologies in immune cell processes, including: Immune cell signaling (TH1/TH2, NK cell, BCR signaling, DC, T-cell receptor signaling pathway) Cytokine signaling (cytokine, IL-12, IL-7 pathway) Immune signal transduction (NFKB, TNF, JAK/STAT pathway)
Mesenchymal (M)	Enriched in: Cell motility (regulation of actin by Rho) ECM receptor interaction Cell differentiation pathways (Wnt/β-catenin, ALK, TGF-β signaling)
Mesenchymal stem-like (MSL)	Similar to M type. Also enriched in: Angiogenesis genes (VEGFR2, TEK, TIE1, EPAS1) Growth factor signaling pathways (including adipocytokine signaling, EGFR, PDGF, G-protein coupled receptor, ERK1/2)
Luminal androgen receptor (LAR)	Enriched in: Signaling pathway of androgen receptor (including FASN, APOD, CLDN8, DHCR24, ALCAM, FKBP5, PIP, SPDEF)

**Table 2:** Role of neoadjuvant carboplatin.

Trial	Design	Treatment	pCR (Carboplatin vs. No Carboplatin)	Survival Outcomes
CALGB-40603 (Alliance) [8,24]	Randomized phase II trial *n* = 443	Weekly paclitaxel plus carboplatin followed by ddAC *w*/*wo* bevacizumab vs. wo carboplatin	60% vs. 44% (*p* = 0.0018)	DFS: HR 0.94; 95% CI 0.67 to 1.32, *p* = 0.72 OS: HR 1.12; 95% CI 0.77 to 1.61, *p* = 0.56
GeparSixto [25,28]	Randomized phase II trial *n* = 315	Paclitaxel plus non-pegylated liposomal doxorubicin plus bevacizumab *w*/*wo* carboplatin	53% vs. 37% (*p* = 0.005)	DFS: HR 0.56; 95% CI 0.34 to 0.93; *p* = 0.022 OS: HR 0.55; 95% CI 0.27 to 1.14, *p* = 0.10
BrighTNess [23,26]	Randomized phase III trial *n* = 634	Paclitaxel vs. paclitaxel plus veliparib plus carboplatin vs. paclitaxel plus carboplatin	31% vs. 53% (*p* < 0.0001) 53% vs. 58% (*p* = 0.36)	EFS: 79.3% vs. 68.5%; HR 0.57, 95% CI 0.36 to 0.91; *p* = 0.018 OS: HR 0.63; 95% CI 0.33 to 1.21, *p* = 0.17
GEICAM/2006–03 [29]	Randomized phase II trial *n* = 94	Epirubicin plus cyclophosphamide followed by docetaxel *w*/*wo* carboplatin	30% vs. 35% (*p* = 0.61)	Not assessed
Ando et al. [30]	Randomized phase II trial *n* = 181	Paclitaxel *w*/*wo* carboplatin followed by cyclophosphamide plus epirubicin and fluorouracil	61.2% vs. 26.3% (*p* = 0.003) in TNBC	Not assessed

Abbreviations: pCR—pathologic complete response, ddAC—dose-dense doxorubicin/cyclophosphamide, DFS—disease-free survival, OS—overall survival, EFS—event-free survival, HR—hazard ratio, CI—confidence interval, TNBC—triple-negative breast cancer, *w*/*wo*—with or without.

**Table 3:** Major clinical trials with chemo-immunotherapy combination in early-stage TNBC.

Neoadjuvant Trials				
Trial	Design	Disease Setting	Treatment	Relevant Endpoint
**Completed trials**				
Impassion031 [47]	Phase III *n* = 455	Neoadjuvant treatment of stage II–III TNBC	Nab-paclitaxel followed by doxorubicin/cyclophosphamide *w*/*wo* atezolizumab	pCR: 58% vs. 41% (rate difference 17%, 95% CI 6 to 27; *p* = 0.0044)
NeoTrip [48]	Phase III *n* = 280	Neoadjuvant treatment of stage II–III TNBC	Carboplatin plus nab-paclitaxel with or without atezolizumab followed by adjuvant anthracycline	pCR: 48.6% vs. 44.4% (OR 1.18, 95% CI 0.74 to 1.89; *p* = 0.48) EFS pending
GeparNuevo [49]	Phase II *n* = 174	Neoadjuvant treatment of cT1b-cT4a-d TNBC	Durvalumab or placebo plus epirubicin/cyclophosphamide	pCR: 53.4% vs. 44.2% (OR 1.45; 95% CI 0.80 to 2.63, *p* = 0.22) 3y DFS: 85.6% vs. 77.2%, *p* = 0.036 OS: 95.2% vs. 83.5%, *p* = 0.006
I-SPY2 [50]	Adaptive Phase II *n* = 250 (29 with TNBC)	Neoadjuvant treatment of high-risk stage II–III breast cancer, including TNBC	Taxane and anthracycline *w*/*wo* pembrolizumab	pCR: 60% vs. 22% in the TNBC cohort
**Ongoing trials**				
GeparDouze [51]	Phase III *n* = 1520	Neoadjuvant plus adjuvant treatment of high-risk (cT2-3N0 or cT1c-3N+) TNBC	NAT with atezolizumab vs. placebo plus paclitaxel/carboplatin followed by AC, six months of postoperative atezolizumab or placebo	EFS, pCR pending
**Adjuvant trials**				
IMpassion030 [52]	Phase III *n* = 2300	Postoperative treatment of operable stage II–III TNBC	Atezolizumab vs. placebo plus anthracycline/taxane	iDFS pending
SWOG S1418 [53]	Phase III *n* = 1155	Postoperative treatment of stage II–III TNBC with residual disease (>1 cm) or lymph node-positive disease (ypN+ including micrometastatic disease) after NAT	12 months of pembrolizumab vs. observation postoperatively	iDFS pending
MIRINAE [54]	Phase II *n* = 284	Postoperative treatment of TNBC with residual disease (>1 cm) or macroscopic positive lymph nodes (ypN+) after NAT	Capecitabine *w*/*wo* atezolizumab	5y iDFS pending

Abbreviations: TNBC—triple-negative breast cancer, EFS—event-free survival, pCR—pathologic complete response, NAT—neoadjuvant therapy, iDFS—invasive disease-free survival, *w*/*wo*—with or without.

**Table 4:** PARPi in early-stage TNBC.

Trial	Trial Characteristics	Setting	Treatment	Result
NEOTALA [69]	Phase II, non-randomized, single arm trial	Early-stage gBRCA1/2-mutated HER2- breast cancer	24 weeks of neoadjuvant talazoparib monotherapy 1 mg daily followed by surgery	pCR was 49%
GeparOLA [70]	Phase II, randomized, open-label trial	Early-stage HER2- breast cancer with either gBRCA1/2 mutation or high HRD score	Neoadjuvant Paclitaxel plus olaparib versus paclitaxel plus carboplatin, both followed by epirubicin/cyclophosphamide	pCR was 55.1% vs. 48.6% In TNBC subgroup, pCR was 56% vs. 59.3%
I-SPY2 [71]	Phase II, adaptive trial	Stage II/III HER2- breast cancer	Neoadjuvant Durvalumab, olaparib, and weekly paclitaxel vs. chemotherapy alone	pCR in the TNBC group higher (27–47%)
I-SPY2 [68]	Phase II, adaptive trial	>2.5 cm stage II/III HER2- breast cancer	Neoadjuvant veliparib with carboplatin plus taxol vs. taxol	pCR rate was higher in the TNBC group at 51% vs. 26%.
BrighTNess [23]	Phase III, randomized, double-blind	Stage II/III TNBC	One of three: Taxol plus carboplatin (AUC6) plus veliparib vs. taxol plus carboplatin (AUC6) plus veliparib placebo vs. taxol plus carboplatin placebo plus veliparib placebo	pCR was 53% vs. 58% vs. 31%

Abbreviations: gBRCA1/2—germline BRCA 1 or 2 mutation, pCR—pathologic complete response, HRD—homologous recombination deficiency, TNBC—triple-negative breast cancer, AUC—area under the curve.

**Table 5:** Ongoing pending trials with targeted treatments in early-stage TNBC.

	Trial	Phase	Setting	Treatment	Primary Endpoint
EGFR	NCT05177796	II	Neoadjuvant inflammatory BC	Panitumumab plus pembrolizumab plus chemotherapy	pCR
FGFR/VEGFR/PDGFR	NCT04427293	I	Neoadjuvant	Lenvatinib plus pembrolizumab	Infiltration of CD8+ TILs
	NeoATCT [NCT04914390]	II	Neoadjuvant	Tislelizumab plus Anlotinib plus anthracycline/nab-paclitaxel	pCR
JAK2/STAT3	NCT02876302	II	Neoadjuvant	Ruxolitinib plus paclitaxel followed by AC	Effect on pStat3+ Expression
	NCT02041429	II	Neoadjuvant inflammatory BC	Ruxolitinib plus paclitaxel followed by AC	Maximum Tolerated Dose
TROP2	NeoSTAR [NCT04230109]	II	Neoadjuvant	Sacituzumab govitecan	pCR rate
	SASCIA [NCT04595565]	III	Adjuvant, HER2- BC with residual disease	Sacituzumab govitecan	iDFS
	ASPRIA [NCT04434040]	II	Adjuvant TNBC with residual disease	Atezolizumab and Sacituzumab govitecan	Undetectable circulating cfDNA
	ASCENT-05 [NCT05633654]	III	Adjuvant TNBC with residual disease	Sacituzumab govitecan plus pembrolizumab vs. pembrolizumab or pembrolizumab/capecitabine	iDFS
	TROPION-Breast03 [NCT05629585]	III	Adjuvant TNBC with residual disease	Datopotamab plus durvalumab vs. Capecitabine *w*/*wo* pembrolizumab	iDFS
CDK4/6	CAREGIVER [NCT05067530]	II	Neoadjuvant	Palbociclib vs. paclitaxel vs. palbociclib plus paclitaxel vs. carboplatin vs. carboplatin plus paclitaxel	Early metabolic response
	NCT03979508	II	Neoadjuvant	NAT, then abemaciclib, then surgery	Change from CD8/FOXP3 ratio <1.6 to >1.6 *

Abbreviations: pCR—pathologic complete response, iDFS—invasive disease-free survival, cfDNA—cell-free DNA, TIL—tumor-infiltrating lymphocytes, NAT—neoadjuvant therapy, *w*/*wo*—with or without; * CD8/FOXP3 ratio is a novel indicator for monitoring immune function. High CD8/FOXP3 ratios are reported to have high pCR rates in TNBC.

## Data Availability

No new data was generated during the preparation of this manuscript.

## References

[1] Dent R., Hanna W.M., Trudeau M., Rawlinson E., Sun P., Narod S.A. (2009). Pattern of Metastatic Spread in Triple-Negative Breast Cancer. Breast Cancer Res. Treat..

[2] Gonzalez-Angulo A.M., Timms K.M., Liu S., Chen H., Litton J.K., Potter J., Lanchbury J.S., Stemke-Hale K., Hennessy B.T., Arun B.K. (2011). Incidence and Outcome of BRCA Mutations in Unselected Patients with Triple Receptor-Negative Breast Cancer. Clin. Cancer Res..

[3] Pereira B., Chin S.-F., Rueda O.M., Vollan H.-K.M., Provenzano E., Bardwell H.A., Pugh M., Jones L., Russell R., Sammut S.-J. (2016). The Somatic Mutation Profiles of 2,433 Breast Cancers Refines Their Genomic and Transcriptomic Landscapes. Nat. Commun..

[4] Shah S.P., Roth A., Goya R., Oloumi A., Ha G., Zhao Y., Turashvili G., Ding J., Tse K., Haffari G. (2012). The Clonal and Mutational Evolution Spectrum of Primary Triple Negative Breast Cancers. Nature.

[5] Bianchini G., Balko J.M., Mayer I.A., Sanders M.E., Gianni L. (2016). Triple-Negative Breast Cancer: Challenges and Opportunities of a Heterogeneous Disease. Nat. Rev. Clin. Oncol..

[6] Perou C.M., Sørlie T., Eisen M.B., van de Rijn M., Jeffrey S.S., Rees C.A., Pollack J.R., Ross D.T., Johnsen H., Akslen L.A. (2000). Molecular Portraits of Human Breast Tumours. Nature.

[7] Sikov W.M., Barry W.T., Hoadley K.A., Pitcher B.N., Singh B., Tolaney S.M., Kuzma C.S., Pluard T.J., Somlo G., Port E.R. (2015). Abstract S4-05: Impact of Intrinsic Subtype by PAM50 and Other Gene Signatures on Pathologic Complete Response (PCR) Rates in Triple-Negative Breast Cancer (TNBC) after Neoadjuvant Chemotherapy (NACT) +/− Carboplatin (Cb) or Bevacizumab (Bev): CALGB 40603/150709 (Allianc). Cancer Res..

[8] Shepherd J.H., Ballman K., Polley M.-Y.C., Campbell J.D., Fan C., Selitsky S., Fernandez-Martinez A., Parker J.S., Hoadley K.A., Hu Z. (2022). CALGB 40603 (Alliance): Long-Term Outcomes and Genomic Correlates of Response and Survival After Neoadjuvant Chemotherapy with or without Carboplatin and Bevacizumab in Triple-Negative Breast Cancer. J. Clin. Oncol..

[9] Lehmann B.D., Pietenpol J.A. (2014). Identification and Use of Biomarkers in Treatment Strategies for Triple-Negative Breast Cancer Subtypes. J. Pathol..

[10] Pinilla K., Drewett L.M., Lucey R., Abraham J.E. (2022). Precision Breast Cancer Medicine: Early Stage Triple Negative Breast Cancer—A Review of Molecular Characterisation, Therapeutic Targets and Future Trends. Front. Oncol..

[11] Liu Y.-R., Jiang Y.-Z., Xu X.-E., Yu K.-D., Jin X., Hu X., Zuo W.-J., Hao S., Wu J., Liu G.-Y. (2016). Comprehensive Transcriptome Analysis Identifies Novel Molecular Subtypes and Subtype-Specific RNAs of Triple-Negative Breast Cancer. Breast Cancer Res..

[12] Rueda O.M., Sammut S.-J., Seoane J.A., Chin S.-F., Caswell-Jin J.L., Callari M., Batra R., Pereira B., Bruna A., Ali H.R. (2019). Dynamics of Breast-Cancer Relapse Reveal Late-Recurring ER-Positive Genomic Subgroups. Nature.

[13] Curtis C., Shah S.P., Chin S.-F., Turashvili G., Rueda O.M., Dunning M.J., Speed D., Lynch A.G., Samarajiwa S., Yuan Y. (2012). The Genomic and Transcriptomic Architecture of 2000 Breast Tumours Reveals Novel Subgroups. Nature.

[14] Prado-Vázquez G., Gámez-Pozo A., Trilla-Fuertes L., Arevalillo J.M., Zapater-Moros A., Ferrer-Gómez M., Díaz-Almirón M., López-Vacas R., Navarro H., Maín P. (2019). A Novel Approach to Triple-Negative Breast Cancer Molecular Classification Reveals a Luminal Immune-Positive Subgroup with Good Prognoses. Sci. Rep..

[15] Burstein M.D., Tsimelzon A., Poage G.M., Covington K.R., Contreras A., Fuqua S.A.W., Savage M.I., Osborne C.K., Hilsenbeck S.G., Chang J.C. (2015). Comprehensive Genomic Analysis Identifies Novel Subtypes and Targets of Triple-Negative Breast Cancer. Clin. Cancer Res..

[16] Symmans W.F., Wei C., Gould R., Yu X., Zhang Y., Liu M., Walls A., Bousamra A., Ramineni M., Sinn B. (2017). Long-Term Prognostic Risk after Neoadjuvant Chemotherapy Associated with Residual Cancer Burden and Breast Cancer Subtype. J. Clin. Oncol..

[17] Thompson A.M., Moulder-Thompson S.L. (2012). Neoadjuvant Treatment of Breast Cancer. Ann. Oncol..

[18] von Minckwitz G., Untch M., Blohmer J.-U., Costa S.D., Eidtmann H., Fasching P.A., Gerber B., Eiermann W., Hilfrich J., Huober J. (2012). Definition and Impact of Pathologic Complete Response on Prognosis after Neoadjuvant Chemotherapy in Various Intrinsic Breast Cancer Subtypes. J. Clin. Oncol..

[19] Cortazar P., Zhang L., Untch M., Mehta K., Costantino J.P., Wolmark N., Bonnefoi H., Cameron D., Gianni L., Vala-gussa P. (2014). Pathological Complete Response and Long-Term Clinical Benefit in Breast Cancer: The CTNeoBC Pooled Analysis. Lancet.

[20] Spring L.M., Fell G., Arfe A., Sharma C., Greenup R., Reynolds K.L., Smith B.L., Alexander B., Moy B., Isakoff S.J. (2020). Pathologic Complete Response after Neoadjuvant Chemotherapy and Impact on Breast Cancer Recurrence and Survival: A Com-prehensive Meta-Analysis. Clin. Cancer Res..

[21] Huang M., O’Shaughnessy J., Zhao J., Haiderali A., Cortes J., Ramsey S., Briggs A., Karantza V., Aktan G., Qi C.Z. (2020). Evaluation of Pathologic Complete Response as a Surrogate for Long-Term Survival Outcomes in Triple-Negative Breast Cancer. J. Natl. Compr. Cancer Netw..

[22] Early Breast Cancer Trialists’ Collaborative Group (EBCTCG) (2023). Anthracycline-Containing and Taxane-Containing Chemotherapy for Early-Stage Operable Breast Cancer: A Patient-Level Meta-Analysis of 100,000 Women from 86 Randomised Trials. Lancet.

[23] Loibl S., O’Shaughnessy J., Untch M., Sikov W.M., Rugo H.S., McKee M.D., Huober J., Golshan M., von Minckwitz G., Maag D. (2018). Addition of the PARP Inhibitor Veliparib plus Carboplatin or Carboplatin Alone to Standard Neoadjuvant Chem-otherapy in Triple-Negative Breast Cancer (BrighTNess): A Randomised, Phase 3 Trial. Lancet Oncol..

[24] Sikov W.M., Berry D.A., Perou C.M., Singh B., Cirrincione C.T., Tolaney S.M., Kuzma C.S., Pluard T.J., Somlo G., Port E.R. (2015). Impact of the Addition of Carboplatin and/or Bevacizumab to Neoadjuvant Once-per-Week Paclitaxel Followed by Dose-Dense Doxorubicin and Cyclophosphamide on Pathologic Complete Response Rates in Stage II to III Triple-Negative Breast Cancer: CALGB 40603 (Alliance). J. Clin. Oncol..

[25] von Minckwitz G., Schneeweiss A., Loibl S., Salat C., Denkert C., Rezai M., Blohmer J.U., Jackisch C., Paepke S., Gerber B. (2014). Neoadjuvant Carboplatin in Patients with Triple-Negative and HER2-Positive Early Breast Cancer (GeparSixto; GBG 66): A Randomised Phase 2 Trial. Lancet Oncol..

[26] Geyer C.E., Sikov W.M., Huober J., Rugo H.S., Wolmark N., O’Shaughnessy J., Maag D., Untch M., Golshan M., Lorenzo J.P. (2022). Long-Term Efficacy and Safety of Addition of Carboplatin with or without Veliparib to Standard Neoadjuvant Chemo-therapy in Triple-Negative Breast Cancer: 4-Year Follow-up Data from BrighTNess, a Randomized Phase III Trial. Ann. Oncol..

[27] Poggio F., Bruzzone M., Ceppi M., Pondé N.F., La Valle G., Del Mastro L., de Azambuja E., Lambertini M. (2018). Platinum-Based Neoadjuvant Chemotherapy in Triple-Negative Breast Cancer: A Systematic Review and Meta-Analysis. Ann. Oncol..

[28] Loibl S., Weber K.E., Timms K.M., Elkin E.P., Hahnen E., Fasching P.A., Lederer B., Denkert C., Schneeweiss A., Braun S. (2018). Survival Analysis of Carboplatin Added to an Anthracycline/Taxane-Based Neoadjuvant Chemotherapy and HRD Score as Predictor of Response-Final Results from GeparSixto. Ann. Oncol..

[29] Alba E., Chacon J.I., Lluch A., Anton A., Estevez L., Cirauqui B., Carrasco E., Calvo L., Segui M.A., Ribelles N. (2012). A Randomized Phase II Trial of Platinum Salts in Basal-like Breast Cancer Patients in the Neoadjuvant Setting. Results from the GEICAM/2006-03, Multicenter Study. Breast Cancer Res. Treat..

[30] Ando M., Yamauchi H., Aogi K., Shimizu S., Iwata H., Masuda N., Yamamoto N., Inoue K., Ohono S., Kuroi K. (2014). Randomized Phase II Study of Weekly Paclitaxel with and without Carboplatin Followed by Cyclophospha-mide/Epirubicin/5-Fluorouracil as Neoadjuvant Chemotherapy for Stage II/IIIA Breast Cancer without HER2 Overexpression. Breast Cancer Res. Treat..

[31] Möbus V., Jackisch C., Lück H.J., du Bois A., Thomssen C., Kuhn W., Nitz U., Schneeweiss A., Huober J., Harbeck N. (2018). Ten-Year Results of Intense Dose-Dense Chemotherapy Show Superior Survival Compared with a Conventional Schedule in High-Risk Primary Breast Cancer: Final Results of AGO Phase III IddEPC Trial. Ann. Oncol..

[32] Early Breast Cancer Trialists’ Collaborative Group (EBCTCG) (2019). Increasing the Dose Intensity of Chemotherapy by More Fre-quent Administration or Sequential Scheduling: A Patient-Level Meta-Analysis of 37,298 Women with Early Breast Cancer in 26 Randomised Trials. Lancet.

[33] Masuda N., Lee S.-J., Ohtani S., Im Y.-H., Lee E.-S., Yokota I., Kuroi K., Im S.-A., Park B.-W., Kim S.-B. (2017). Adjuvant Capecitabine for Breast Cancer after Preoperative Chemotherapy. N. Engl. J. Med..

[34] Wang X., Wang S.-S., Huang H., Cai L., Zhao L., Peng R.-J., Lin Y., Tang J., Zeng J., Zhang L.-H. (2021). Effect of Capecitabine Maintenance Therapy Using Lower Dosage and Higher Frequency vs. Observation on Disease-Free Survival Among Patients With Early-Stage Triple-Negative Breast Cancer Who Had Received Standard Treatment: The SYSUCC-001 Randomized Clinical Trial. JAMA.

[35] Lluch A., Barrios C.H., Torrecillas L., Ruiz-Borrego M., Bines J., Segalla J., Guerrero-Zotano Á., García-Sáenz J.A., Torres R., de la Haba J. (2020). Phase III Trial of Adjuvant Capecitabine After Standard Neo-/Adjuvant Chemotherapy in Patients With Early Triple-Negative Breast Cancer (GEICAM/2003-11_CIBOMA/2004-01). J. Clin. Oncol..

[36] García-Teijido P., Cabal M.L., Fernández I.P., Pérez Y.F. (2016). Tumor-Infiltrating Lymphocytes in Triple Negative Breast Cancer: The Future of Immune Targeting. Clin. Med. Insights Oncol..

[37] Stanton S.E., Adams S., Disis M.L. (2016). Variation in the Incidence and Magnitude of Tumor-Infiltrating Lymphocytes in Breast Cancer Subtypes: A Systematic Review. JAMA Oncol..

[38] Thomas A., Routh E.D., Pullikuth A., Jin G., Su J., Chou J.W., Hoadley K.A., Print C., Knowlton N., Black M.A. (2018). Tumor Mutational Burden Is a Determinant of Immune-Mediated Survival in Breast Cancer. Oncoimmunology.

[39] Wimberly H., Brown J.R., Schalper K., Haack H., Silver M.R., Nixon C., Bossuyt V., Pusztai L., Lannin D.R., Rimm D.L. (2015). PD-L1 Expression Correlates with Tumor-Infiltrating Lymphocytes and Response to Neoadjuvant Chemotherapy in Breast Cancer. Cancer Immunol. Res..

[40] O’Meara T.A., Tolaney S.M. (2021). Tumor Mutational Burden as a Predictor of Immunotherapy Response in Breast Cancer. Oncotarget.

[41] Cortes J., Cescon D.W., Rugo H.S., Nowecki Z., Im S.-A., Yusof M.M., Gallardo C., Lipatov O., Barrios C.H., Holgado E. (2020). Pembrolizumab plus Chemotherapy versus Placebo plus Chemotherapy for Previously Untreated Locally Recurrent Inoperable or Metastatic Triple-Negative Breast Cancer (KEYNOTE-355): A Randomised, Placebo-Controlled, Double-Blind, Phase 3 Clinical Trial. Lancet.

[42] Schmid P., Adams S., Rugo H.S., Schneeweiss A., Barrios C.H., Iwata H., Diéras V., Hegg R., Im S.-A., Shaw Wright G. (2018). Atezolizumab and Nab-Paclitaxel in Advanced Triple-Negative Breast Cancer. N. Engl. J. Med..

[43] Hutchinson K.E., Yost S.E., Chang C.-W., Johnson R.M., Carr A.R., McAdam P.R., Halligan D.L., Chang C.-C., Schmolze D., Liang J. (2020). Comprehensive Profiling of Poor-Risk Paired Primary and Recurrent Triple-Negative Breast Cancers Reveals Immune Phenotype Shifts. Clin. Cancer Res..

[44] Szekely B., Bossuyt V., Li X., Wali V.B., Patwardhan G.A., Frederick C., Silber A., Park T., Harigopal M., Pelekanou V. (2018). Immunological Differences between Primary and Metastatic Breast Cancer. Ann. Oncol..

[45] Schmid P., Cortes J., Pusztai L., McArthur H., Kümmel S., Bergh J., Denkert C., Park Y.H., Hui R., Harbeck N. (2020). Pembrolizumab for Early Triple-Negative Breast Cancer. N. Engl. J. Med..

[46] Schmid P., Cortes J., Dent R., Pusztai L., McArthur H., Kümmel S., Bergh J., Denkert C., Park Y.H., Hui R. (2022). Event-Free Survival with Pembrolizumab in Early Triple-Negative Breast Cancer. N. Engl. J. Med..

[47] Mittendorf E.A., Zhang H., Barrios C.H., Saji S., Jung K.H., Hegg R., Koehler A., Sohn J., Iwata H., Telli M.L. (2020). Neoadjuvant Atezolizumab in Combination with Sequential Nab-Paclitaxel and Anthracycline-Based Chemotherapy versus Pla-cebo and Chemotherapy in Patients with Early-Stage Triple-Negative Breast Cancer (IMpassion031): A Randomised, Double-Blind, Phase 3 Trial. Lancet.

[48] Gianni L., Huang C.S., Egle D., Bermejo B., Zamagni C., Thill M., Anton A., Zambelli S., Bianchini G., Russo S. (2022). Pathologic Complete Response (PCR) to Neoadjuvant Treatment with or without Atezolizumab in Triple-Negative, Early High-Risk and Locally Advanced Breast Cancer: NeoTRIP Michelangelo Randomized Study. Ann. Oncol..

[49] Loibl S., Schneeweiss A., Huober J., Braun M., Rey J., Blohmer J.-U., Furlanetto J., Zahm D.-M., Hanusch C., Thomalla J. (2022). Neoadjuvant Durvalumab Improves Survival in Early Triple-Negative Breast Cancer Independent of Pathological Complete Response. Ann. Oncol..

[50] Nanda R., Liu M.C., Yau C., Shatsky R., Pusztai L., Wallace A., Chien A.J., Forero-Torres A., Ellis E., Han H. (2020). Effect of Pembrolizumab Plus Neoadjuvant Chemotherapy on Pathologic Complete Response in Women with Early-Stage Breast Cancer: An Analysis of the Ongoing Phase 2 Adaptively Randomized I-SPY2 Trial. JAMA Oncol..

[51] Loibl S., Jackisch C., Rastogi P., Seiler S., Lucas P.C., Denkert C., Costantino J., Nekljudova V., Wolmark N., Geyer C. (2019). GeparDouze/NSABP B-59: A Randomized Double-Blind Phase III Clinical Trial of Neoadjuvant Chemotherapy with Atezolizumab or Placebo in Patients with Triple Negative Breast Cancer (TNBC) Followed by Adjuvant Atezolizumab or Placebo. Ann. Oncol..

[52] Saji S., McArthur H.L., Ignatiadis M., Bailey A., El-Abed S., Brandao M., Metzger O., Lai C., Guillaume S., Fumagalli D. (2021). ALEXANDRA/IMpassion030: A Phase 3 Study of Standard Adjuvant Chemotherapy with or without Atezolizumab in Pa-tients with Early-Stage Triple-Negative Breast Cancer. J. Clin. Oncol..

[53] Abstract OT1-02-04: SWOG S1418/NRG-BR006: A Randomized, Phase III Trial to Evaluate the Efficacy and Safety of MK-3475 as Adjuvant Therapy for Triple Receptor-Negative Breast Cancer with >1 cm Residual Invasive Cancer or Positive Lymph Nodes (>pN1mic) after Neoadjuvant Chemotherapy|Cancer Research|American Association for Cancer Research. https://aacrjournals.org/cancerres/article/78/4_Supplement/OT1-02-04/632005/Abstract-OT1-02-04-SWOG-S1418-NRG-BR006-A.

[54] Park I.H., Kim G.M., Kim J.H., Kim H., Park K.H., Park Y.H., Baek S.K., Sim S.H., Ahn H.K., Lee G.-W. (2020). Ran-domized, Phase II Trial to Evaluate the Efficacy and Safety of Atezolizumab plus Capecitabine Adjuvant Therapy Compared to Capecitabine Monotherapy for Triple Receptor-Negative Breast Cancer (TNBC) with Residual Invasive Cancer after Neoadjuvant Chemotherapy (MIRINAE Trial, KCSG-BR18-21). J. Clin. Oncol..

[55] Zhang Q., Luo J., Wu S., Si H., Gao C., Xu W., Abdullah S.E., Higgs B.W., Dennis P.A., van der Heijden M.S. (2020). Prognostic and Predictive Impact of Circulating Tumor DNA in Patients with Advanced Cancers Treated with Immune Checkpoint Blockade. Cancer Discov..

[56] Lu C., Zhang Y.-C., Chen Z.-H., Zhou Q., Wu Y.-L. (2022). Applications of Circulating Tumor DNA in Immune Checkpoint Inhibition: Emerging Roles and Future Perspectives. Front. Oncol..

[57] Tutt A.N.J., Garber J.E., Kaufman B., Viale G., Fumagalli D., Rastogi P., Gelber R.D., de Azambuja E., Fielding A., Balmaña J. (2021). Adjuvant Olaparib for Patients with *BRCA1*- or *BRCA2*-Mutated Breast Cancer. N. Engl. J. Med..

[58] Tarantino P., Corti C., Schmid P., Cortes J., Mittendorf E.A., Rugo H., Tolaney S.M., Bianchini G., Andrè F., Curigliano G. (2022). Immunotherapy for Early Triple Negative Breast Cancer: Research Agenda for the next Decade. NPJ Breast Cancer.

[59] Hirsch I., Goldstein D.A., Tannock I.F., Butler M.O., Gilbert D.C. (2022). Optimizing the Dose and Schedule of Immune Checkpoint Inhibitors in Cancer to Allow Global Access. Nat. Med..

[60] Santa-Maria C.A. (2023). Optimizing and Refining Immunotherapy in Breast Cancer. JCO Oncol. Pract..

[61] Pusztai L., Denkert C., O’Shaughnessy J., Cortes J., Dent R.A., McArthur H.L., Kuemmel S., Bergh J.C.S., Park Y.H., Hui R. (2022). Event-Free Survival by Residual Cancer Burden after Neoadjuvant Pembrolizumab + Chemotherapy versus Placebo + Chemotherapy for Early TNBC: Exploratory Analysis from KEYNOTE-522. J. Clin. Oncol..

[62] German Breast Group (2023). Phase III Postneoadjuvant Study Evaluating Sacituzumab Govitecan, an Antibody Drug Conjugate in Primary HER2-Negative Breast Cancer Patients with High Relapse Risk after Standard Neoadjuvant Treatment—SASCIA. https://www.clinicaltrials.gov/study/NCT04595565?cond=Sacituzumab%20Govitecan%20in%20Primary%20HER2-negative%20Breast%20Cancer%20(SASCIA)&rank=1.

[63] Gilead Sciences (2023). A Randomized, Open-Label, Phase 3 Study of Adjuvant Sacituzumab Govitecan and Pembrolizumab Versus Treatment of Physician’s Choice in Patients with Triple Negative Breast Cancer Who Have Residual Invasive Disease after Surgery and Neoadjuvant Therapy. https://www.clinicaltrials.gov/study/NCT05633654?cond=Who%20Have%20Residual%20Invasive%20Disease%20after%20Surgery%20and%20Neoadjuvant%20Therapy&rank=1.

[64] Abdou Y., Goudarzi A., Yu J.X., Upadhaya S., Vincent B., Carey L.A. (2022). Immunotherapy in Triple Negative Breast Cancer: Beyond Checkpoint Inhibitors. NPJ Breast Cancer.

[65] Soliman H., Hogue D., Han H., Mooney B., Costa R., Lee M.C., Niell B., Williams A., Chau A., Falcon S. (2023). Oncolytic T-VEC Virotherapy plus Neoadjuvant Chemotherapy in Nonmetastatic Triple-Negative Breast Cancer: A Phase 2 Trial. Nat. Med..

[66] Barchiesi G., Roberto M., Verrico M., Vici P., Tomao S., Tomao F. (2021). Emerging Role of PARP Inhibitors in Metastatic Triple Negative Breast Cancer. Current Scenario and Future Perspectives. Front. Oncol..

[67] Geyer C.E., Garber J.E., Gelber R.D., Yothers G., Taboada M., Ross L., Rastogi P., Cui K., Arahmani A., Aktan G. (2022). Overall Survival in the OlympiA Phase III Trial of Adjuvant Olaparib in Patients with Germline Pathogenic Variants in BRCA1/2 and High-Risk, Early Breast Cancer. Ann. Oncol..

[68] Rugo H.S., Olopade O.I., DeMichele A., Yau C., van ’t Veer L.J., Buxton M.B., Hogarth M., Hylton N.M., Paoloni M., Perlmutter J. (2016). Adaptive Randomization of Veliparib–Carboplatin Treatment in Breast Cancer. N. Engl. J. Med..

[69] Litton J.K., Scoggins M.E., Hess K.R., Adrada B.E., Murthy R.K., Damodaran S., DeSnyder S.M., Brewster A.M., Bar-cenas C.H., Valero V. (2020). Neoadjuvant Talazoparib for Patients with Operable Breast Cancer with a Germline BRCA Pathogenic Variant. J. Clin. Oncol..

[70] Fasching P.A., Link T., Hauke J., Seither F., Jackisch C., Klare P., Schmatloch S., Hanusch C., Huober J., Stefek A. (2021). Neoadjuvant Paclitaxel/Olaparib in Comparison to Paclitaxel/Carboplatinum in Patients with HER2-Negative Breast Cancer and Homologous Recombination Deficiency (GeparOLA Study). Ann. Oncol..

[71] Pusztai L., Yau C., Wolf D.M., Han H.S., Du L., Wallace A.M., String-Reasor E., Boughey J.C., Chien A.J., Elias A.D. (2021). Durvalumab with Olaparib and Paclitaxel for High-Risk HER2-Negative Stage II/III Breast Cancer: Results from the Adaptively Randomized I-SPY2 Trial. Cancer Cell.

[72] Jiang Y.-Z., Ma D., Suo C., Shi J., Xue M., Hu X., Xiao Y., Yu K.-D., Liu Y.-R., Yu Y. (2019). Genomic and Transcriptomic Landscape of Triple-Negative Breast Cancers: Subtypes and Treatment Strategies. Cancer Cell.

[73] Koboldt D.C., Fulton R.S., McLellan M.D., Schmidt H., Kalicki-Veizer J., McMichael J.F., Fulton L.L., Dooling D.J., Ding L., Mardis E.R. (2012). Comprehensive Molecular Portraits of Human Breast Tumours. Nature.

[74] Montero J.C., Esparís-Ogando A., Re-Louhau M.F., Seoane S., Abad M., Calero R., Ocaña A., Pandiella A. (2014). Active Kinase Profiling, Genetic and Pharmacological Data Define MTOR as an Important Common Target in Triple-Negative Breast Cancer. Oncogene.

[75] Oliveira M., Saura C., Nuciforo P., Calvo I., Andersen J., Passos-Coelho J.L., Gil Gil M., Bermejo B., Patt D.A., Ciruelos E. (2019). FAIRLANE, a Double-Blind Placebo-Controlled Randomized Phase II Trial of Neoadjuvant Ipatasertib plus Paclitaxel for Early Triple-Negative Breast Cancer. Ann. Oncol..

[76] Jovanović B., Mayer I.A., Mayer E.L., Abramson V.G., Bardia A., Sanders M.E., Kuba M.G., Estrada M.V., Beeler J.S., Shaver T.M. (2017). A Randomized Phase II Neoadjuvant Study of Cisplatin, Paclitaxel with or without Everolimus in Patients with Stage II/III Triple-Negative Breast Cancer (TNBC): Responses and Long-Term Outcome Correlated with Increased Frequency of DNA Damage Response Gene Mutations, TNBC Subtype, AR Status, and Ki67. Clin. Cancer Res..

[77] Gonzalez-Angulo A.M., Akcakanat A., Liu S., Green M.C., Murray J.L., Chen H., Palla S.L., Koenig K.B., Brewster A.M., Valero V. (2014). Open-Label Randomized Clinical Trial of Standard Neoadjuvant Chemotherapy with Paclitaxel Followed by FEC versus the Combination of Paclitaxel and Everolimus Followed by FEC in Women with Triple Receptor-Negative Breast Cancer^†^. Ann. Oncol..

[78] Park S., Koo J., Park H.S., Kim J.-H., Choi S.-Y., Lee J.H., Park B.-W., Lee K.S. (2010). Expression of Androgen Receptors in Primary Breast Cancer. Ann. Oncol..

[79] Traina T.A., Miller K., Yardley D.A., Eakle J., Schwartzberg L.S., O’Shaughnessy J., Gradishar W., Schmid P., Winer E., Kelly C. (2018). Enzalutamide for the Treatment of Androgen Receptor–Expressing Triple-Negative Breast Cancer. J. Clin. Oncol..

[80] Lehmann B.D., Abramson V.G., Sanders M.E., Mayer E.L., Haddad T.C., Nanda R., Van Poznak C., Storniolo A.M., Nangia J.R., Gonzalez-Ericsson P.I. (2020). TBCRC 032 IB/II Multicenter Study: Molecular Insights to AR Antagonist and PI3K Inhibitor Efficacy in Patients with AR^+^ Metastatic Triple-Negative Breast Cancer. Clin. Cancer Res..

[81] Bonnefoi H., Grellety T., Tredan O., Saghatchian M., Dalenc F., Mailliez A., L’Haridon T., Cottu P., Abadie-Lacourtoisie S., You B. (2016). A Phase II Trial of Abiraterone Acetate plus Prednisone in Patients with Triple-Negative Androgen Receptor Positive Locally Advanced or Metastatic Breast Cancer (UCBG 12-1). Ann. Oncol..

[82] Gucalp A., Tolaney S., Isakoff S.J., Ingle J.N., Liu M.C., Carey L.A., Blackwell K., Rugo H., Nabell L., Forero A. (2013). Phase II Trial of Bicalutamide in Patients with Androgen Receptor-Positive, Estrogen Receptor-Negative Metastatic Breast Cancer. Clin. Cancer Res..

[83] Wulfkuhle J.D., Yau C., Wolf D.M., Vis D.J., Gallagher R.I., Brown-Swigart L., Hirst G., Voest E.E., DeMichele A., Hylton N. (2018). Evaluation of the HER/PI3K/AKT Family Signaling Network as a Predictive Biomarker of Pathologic Complete Response for Patients with Breast Cancer Treated with Neratinib in the I-SPY 2 TRIAL. JCO Precis. Oncol..

[84] Wulfkuhle J.D., Yau C., Wolf D.M., Gallagher R.I., Deng J., Brown Swigart L., Hirst G., Liu M.C., Park J.W., Esserman L. (2015). Protein Activation Mapping and Exploratory Predictive Markers for PCR in Triple-Negative Breast Cancer Patients Treated with Neratinib in the I-SPY 2 TRIAL. J. Clin. Oncol..

[85] Schettini F., Chic N., Brasó-Maristany F., Paré L., Pascual T., Conte B., Martínez-Sáez O., Adamo B., Vidal M., Barnadas E. (2021). Clinical, Pathological, and PAM50 Gene Expression Features of HER2-Low Breast Cancer. NPJ Breast Cancer.

[86] Modi S., Jacot W., Yamashita T., Sohn J., Vidal M., Tokunaga E., Tsurutani J., Ueno N.T., Prat A., Chae Y.S. (2022). Trastuzumab Deruxtecan in Previously Treated HER2-Low Advanced Breast Cancer. N. Engl. J. Med..

[87] Earl H.M., Hiller L., Dunn J.A., Blenkinsop C., Grybowicz L., Vallier A.-L., Abraham J., Thomas J., Provenzano E., Hughes-Davies L. (2015). Efficacy of Neoadjuvant Bevacizumab Added to Docetaxel Followed by Fluorouracil, Epirubicin, and Cyclophosphamide, for Women with HER2-Negative Early Breast Cancer (ARTemis): An Open-Label, Randomised, Phase 3 Trial. Lancet Oncol..

[88] Earl H.M., Hiller L., Dunn J.A., Blenkinsop C., Grybowicz L., Vallier A.-L., Gounaris I., Abraham J.E., Hughes-Davies L., McAdam K. (2017). Disease-Free and Overall Survival at 3.5 Years for Neoadjuvant Bevacizumab Added to Docetaxel Followed by Fluorouracil, Epirubicin and Cyclophosphamide, for Women with HER2 Negative Early Breast Cancer: ARTemis Trial. Ann. Oncol..

[89] Cameron D., Brown J., Dent R., Jackisch C., Mackey J., Pivot X., Steger G.G., Suter T.M., Toi M., Parmar M. (2013). Adjuvant Bevacizumab-Containing Therapy in Triple-Negative Breast Cancer (BEATRICE): Primary Results of a Randomised, Phase 3 Trial. Lancet Oncol..

[90] Colavito S.A. (2020). AXL as a Target in Breast Cancer Therapy. J. Oncol..

[91] Ebrahimi N., Kharazmi K., Ghanaatian M., Miraghel S.A., Amiri Y., Seyedebrahimi S.S., Mobarak H., Yazdani E., Parkhideh S., Hamblin M.R. (2022). Role of the Wnt and GTPase Pathways in Breast Cancer Tumorigenesis and Treatment. Cytokine Growth Factor Rev..

[92] Campagna R., Pozzi V., Giorgini S., Morichetti D., Goteri G., Sartini D., Serritelli E.N., Emanuelli M. (2023). Paraoxonase-2 Is Upregulated in Triple Negative Breast Cancer and Contributes to Tumor Progression and Chemoresistance. Hum. Cell.

[93] Spanheimer P.M., Carr J.C., Thomas A., Sugg S.L., Scott-Conner C.E., Liao J., Weigel R.J. (2013). The Response to Neoadjuvant Chemotherapy Predicts Clinical Outcome and Increases Breast Conservation in Advanced Breast Cancer. Am. J. Surg..

[94] Golshan M., Loibl S., Wong S.M., Houber J.B., O’Shaughnessy J., Rugo H.S., Wolmark N., McKee M.D., Maag D., Sul-livan D.M. (2020). Breast Conservation after Neoadjuvant Chemotherapy for Triple-Negative Breast Cancer. JAMA Surg..

[95] Donker M., van Tienhoven G., Straver M.E., Meijnen P., van de Velde C.J.H., Mansel R.E., Cataliotti L., Westenberg A.H., Klinkenbijl J.H.G., Orzalesi L. (2014). Radiotherapy or Surgery of the Axilla after a Positive Sentinel Node in Breast Cancer (EORTC 10981-22023 AMAROS): A Randomised, Multicentre, Open-Label, Phase 3 Non-Inferiority Trial. Lancet Oncol..

[96] Fleissig A., Fallowfield L.J., Langridge C.I., Johnson L., Newcombe R.G., Dixon J.M., Kissin M., Mansel R.E. (2006). Post-Operative Arm Morbidity and Quality of Life. Results of the ALMANAC Randomised Trial Comparing Sentinel Node Biopsy with Standard Axillary Treatment in the Management of Patients with Early Breast Cancer. Breast Cancer Res. Treat..

[97] Boughey J.C., Suman V.J., Mittendorf E.A., Ahrendt G.M., Wilke L.G., Taback B., Leitch A.M., Kuerer H.M., Bowling M., Flippo-Morton T.S. (2013). Sentinel Lymph Node Surgery after Neoadjuvant Chemotherapy in Patients with Node-Positive Breast Cancer: The ACOSOG Z1071 (Alliance) Clinical Trial. JAMA.

[98] Kuehn T., Bauerfeind I., Fehm T., Fleige B., Hausschild M., Helms G., Lebeau A., Liedtke C., von Minckwitz G., Nekljudova V. (2013). Sentinel-Lymph-Node Biopsy in Patients with Breast Cancer before and after Neoadjuvant Chemotherapy (SENTINA): A Prospective, Multicentre Cohort Study. Lancet Oncol..

[99] Caudle A.S., Yang W.T., Krishnamurthy S., Mittendorf E.A., Black D.M., Gilcrease M.Z., Bedrosian I., Hobbs B.P., DeSnyder S.M., Hwang R.F. (2016). Improved Axillary Evaluation Following Neoadjuvant Therapy for Patients With Node-Positive Breast Cancer Using Selective Evaluation of Clipped Nodes: Implementation of Targeted Axillary Dissection. J. Clin. Oncol..

[100] Gallagher K.K., Iles K., Kuzmiak C., Louie R., McGuire K.P., Ollila D.W. (2022). Prospective Evaluation of Radar-Localized Re-flector-Directed Targeted Axillary Dissection in Node-Positive Breast Cancer Patients after Neoadjuvant Systemic Therapy. J. Am. Coll. Surg..

[101] Barrio A.V., Montagna G., Mamtani A., Sevilimedu V., Edelweiss M., Capko D., Cody H.S., El-Tamer M., Gemignani M.L., Heerdt A. (2021). Nodal Recurrence in Patients with Node-Positive Breast Cancer Treated with Sentinel Node Biopsy Alone after Neoadjuvant Chemotherapy-A Rare Event. JAMA Oncol..

[102] Giuliano A.E., Ballman K.V., McCall L., Beitsch P.D., Brennan M.B., Kelemen P.R., Ollila D.W., Hansen N.M., Whitworth P.W., Blumencranz P.W. (2017). Effect of Axillary Dissection vs No Axillary Dissection on 10-Year Overall Survival Among Women with Invasive Breast Cancer and Sentinel Node Metastasis. JAMA.

[103] NSABP Foundation Inc (2022). A Randomized Phase III Clinical Trial Evaluating Post-Mastectomy Chestwall and Regional Nodal XRT and Post-Lumpectomy Regional Nodal XRT in Patients With Positive Axillary Nodes Before Neoadjuvant Chemotherapy Who Convert to Pathologically Negative Axillary Nodes after Neoadjuvant Chemotherapy. https://www.clinicaltrials.gov/study/NCT01872975?cond=Phase%20III%20Clinical%20Trial%20Evaluating%20Post-Mastectomy%20Chestwall%20and%20Regional%20Nodal%20XRT%20&rank=1.

[104] Kuerer H.M., Smith B.D., Krishnamurthy S., Yang W.T., Valero V., Shen Y., Lin H., Lucci A., Boughey J.C., White R.L. (2022). Eliminating Breast Surgery for Invasive Breast Cancer in Exceptional Responders to Neoadjuvant Systemic Therapy: A Mul-ticentre, Single-Arm, Phase 2 Trial. Lancet Oncol..

[105] Yin L., Duan J.-J., Bian X.-W., Yu S. (2020). Triple-Negative Breast Cancer Molecular Subtyping and Treatment Progress. Breast Cancer Res..

[106] Dagogo-Jack I., Shaw A.T. (2018). Tumour Heterogeneity and Resistance to Cancer Therapies. Nat. Rev. Clin. Oncol..

[107] Yu C., Mannan A.M., Yvone G.M., Ross K.N., Zhang Y.-L., Marton M.A., Taylor B.R., Crenshaw A., Gould J.Z., Tamayo P. (2016). High-Throughput Identification of Genotype-Specific Cancer Vulnerabilities in Mixtures of Barcoded Tumor Cell Lines. Nat. Biotechnol..

[108] Tsherniak A., Vazquez F., Montgomery P.G., Weir B.A., Kryukov G., Cowley G.S., Gill S., Harrington W.F., Pantel S., Krill-Burger J.M. (2017). Defining a Cancer Dependency Map. Cell.

[109] Shu S., Wu H.-J., Ge J.Y., Zeid R., Harris I.S., Jovanović B., Murphy K., Wang B., Qiu X., Endress J.E. (2020). Synthetic Lethal and Resistance Interactions with BET Bromodomain Inhibitors in Triple-Negative Breast Cancer. Mol. Cell.

[110] Rashid N.S., Hairr N.S., Murray G., Olex A.L., Leftwich T.J., Grible J.M., Reed J., Dozmorov M.G., Harrell J.C. (2021). Identi-fication of Nuclear Export Inhibitor-Based Combination Therapies in Preclinical Models of Triple-Negative Breast Cancer. Transl. Oncol..

[111] Gambardella G., Viscido G., Tumaini B., Isacchi A., Bosotti R., di Bernardo D. (2022). A Single-Cell Analysis of Breast Cancer Cell Lines to Study Tumour Heterogeneity and Drug Response. Nat. Commun..

[112] Wu S.Z., Al-Eryani G., Roden D.L., Junankar S., Harvey K., Andersson A., Thennavan A., Wang C., Torpy J.R., Bar-tonicek N. (2021). A Single-Cell and Spatially Resolved Atlas of Human Breast Cancers. Nat. Genet..

[113] Paik S., Tang G., Shak S., Kim C., Baker J., Kim W., Cronin M., Baehner F.L., Watson D., Bryant J. (2006). Gene Expression and Benefit of Chemotherapy in Women with Node-Negative, Estrogen Receptor-Positive Breast Cancer. J. Clin. Oncol..

[114] Paik S., Shak S., Tang G., Kim C., Baker J., Cronin M., Baehner F.L., Walker M.G., Watson D., Park T. (2004). A Multigene Assay to Predict Recurrence of Tamoxifen-Treated, Node-Negative Breast Cancer. N. Engl. J. Med..

[115] Parker J.S., Mullins M., Cheang M.C.U., Leung S., Voduc D., Vickery T., Davies S., Fauron C., He X., Hu Z. (2009). Su-pervised Risk Predictor of Breast Cancer Based on Intrinsic Subtypes. J. Clin. Oncol..

[116] Zhang Y., Chen H., Mo H., Hu X., Gao R., Zhao Y., Liu B., Niu L., Sun X., Yu X. (2021). Single-Cell Analyses Reveal Key Immune Cell Subsets Associated with Response to PD-L1 Blockade in Triple-Negative Breast Cancer. Cancer Cell.

